# Smart traffic management of vehicles using faster R-CNN based deep learning method

**DOI:** 10.1038/s41598-024-60596-4

**Published:** 2024-05-06

**Authors:** Arindam Chaudhuri

**Affiliations:** Great Lakes Institute of Management, Chennai, India

**Keywords:** Smart traffic management, Vehicle segmentation, Traffic density estimation, Vehicle tracking, Faster R-CNN, Engineering, Mathematics and computing

## Abstract

With constant growth of civilization and modernization of cities all across the world since past few centuries smart traffic management of vehicles is one of the most sorted after problem by research community. Smart traffic management basically involves segmentation of vehicles, estimation of traffic density and tracking of vehicles. The vehicle segmentation from videos helps realization of niche applications such as monitoring of speed and estimation of traffic. When occlusions, background with clutters and traffic with density variations, this problem becomes more intractable in nature. Keeping this motivation in this research work, we investigate Faster R-CNN based deep learning method towards segmentation of vehicles. This problem is addressed in four steps viz minimization with adaptive background model, Faster R-CNN based subnet operation, Faster R-CNN initial refinement and result optimization with extended topological active nets. The computational framework uses adaptive background modeling. It also addresses shadow and illumination issues. Higher segmentation accuracy is achieved through topological active net deformable models. The topological and extended topological active nets help to achieve stated deformations. Mesh deformation is achieved with minimization of energy. The segmentation accuracy is improved with modified version of extended topological active net. The experimental results demonstrate superiority of this framework with respect to other methods.

## Introduction

We are facing the challenge of continuous increase in traffic density across different cities of world. The present world population is heavily dependent on vehicles from smooth commutation purposes. This results from constant urbanization of rural areas. In order to address this problem, smart traffic management is studied by researchers as traffic regulations solution.

**Figure 1:**
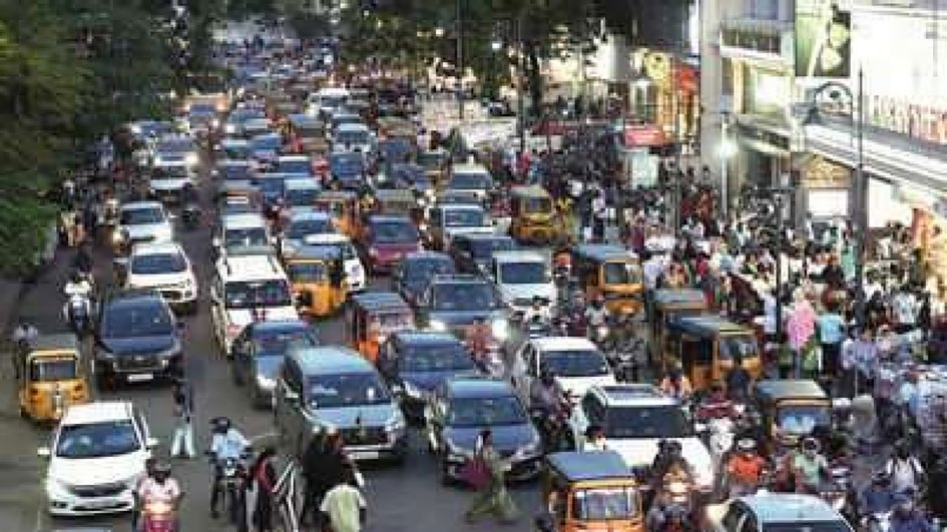
A real life crowded place from Indian traffic.

Human vision system has the capability to perform complex tasks very reliably and accurately. Humans detect wide spectrum of objects very easily. With recent developments in computer vision coupled with huge data sets, better algorithms and faster GPUs, precision and accuracy of object detection and classification algorithms^1,2^ have increased to an appreciable amount. For traffic monitoring vehicle localization efficiency is very important. The vehicles from Indian traffic are presented in Fig. 1. For public safety autonomous vehicle detection methods^3^ are in place which detect traffic objects in order to achieve correct decisions. Smart traffic management allows us to take care of various traffic related issues in an optimum manner. This is achieved using computer vision and image processing. Segmentation of vehicle acts as significant enabler in smart traffic management functionality. With occlusions, congestion as well as other environmental factors this problem becomes more severe. For smooth and effective traffic regulations, smart traffic management is always considered as low cost solution. Congestions, emergency vehicles transport, accidents and traffic related violations are easily managed through latest Artificial Intelligence algorithms. Vehicle segmentation is an important area towards smart traffic management. Some other activities here include traffic estimation, speed control and vehicle tracking. During occlusions, fog, haze, clutters and heavy traffic situations things become more complicated.

With the growth of deep learning networks vehicle detection is being studied deeply considering traffic congestion and driving safety^4^. Vehicle localization is a crucial problem^5^ in order to develop intelligent and autonomous systems. The detection of abnormalities arising from traffic violations leads to the problem of vehicle localization. This is a significant application catering the needs for variety of traffic related problems. The traffic surveillance has been a major concern in densely populated geographical areas. These days’ surveillance systems are well equipped with traffic flow data where various traffic patterns are recorded. Some notable applications here include many smart cities based applications. Different versions of deep learning network based methods are in place which have provided considerable benefits for these applications^3^.

In this research work, we investigate Faster R-CNN based deep learning method towards segmentation of vehicles. This problem is addressed in four steps^5^: (a) minimization with adaptive background model (b) Faster R-CNN based subnet operation (c) Faster R-CNN initial refinement and (d) result optimization with extended topological active nets. Adaptive gain function is used adaptive background modeling. The gain function compensates for shadow and illumination issues. Higher accuracy in segmentation is provided by topological active net deformable model in various situations. Deformable models provide various curvatures with respect to image surfaces. The smoothness from deformation is achieved through several forces considering objects of interest. The topological and extended topological active nets are used here in order to achieve stated deformations. This helps in fitting objects on 2D surface mesh of image. The deformation of mesh is achieved with minimization of energy. For problems with complex shapes energy is changed through extended topological active net with certain thresholds. The segmentation accuracy is increased with improved version of extended topological active net. This solution has combined effect from all models using which better segmentation boundaries are achieved. The performance of stated method is compared with respect to some important metrics such as accuracy, specificity, recall, precision and F1-score values as well as Type I and Type II errors. This method provides better segmentation performance in comparison to other methods. The experimental hypothesis is justified with benchmarked datasets. The model provides appreciable results for different sets of image datasets. This research work has been approved by Research and Development Review Board at Samsung R&D Institute Delhi. This chapter is structured as follows. The literature review is presented in “Literature review” section. In “Computational method” section highlights detailed discussion on computational method. The simulation results are shown in “Experiments and results” section. Finally, conclusion is provided in “Conclusion” section.

## Literature review

During recent decades’ smart traffic management involving vehicle detection has gained considerable attention. Some of the notable works are discussed here. In Ref.^6^ a pixel wise classification method based on dynamic bayesian network for vehicle detection is proposed. In Ref.^7^ an object detection scheme is presented which identifies changes in image series. Foreground vehicle segmentation using gaussian mixture model is highlighted in Ref.^8^. An adaptive background model having frames averaging with respect to time is discussed in Ref.^9^. For vehicle localization ResNet model is used in Ref.^10^. A vehicle classification system involving deep learning is presented in Ref.^11^. An integrated vehicle detection and classification method is discussed in Ref.^12^. Song et al.^13^discusses YOLOv3 algorithm for vehicle detection. Lee et al.^14^ presents receptive field based neural network. Semantic image segmentation is used in Ref.^15^. Mask R-CNN with transfer learning is used in Ref.^16^. Shan et al.^17^discusses YOLO based solution. A review on vehicle detection is highlighted in Ref.^18^. In Ref.^19^ deep learning assisted vehicle segmentation is discussed.

In Ref.^20^ vehicles in airborne images are detected. An ensemble based method using image descriptors is discussed in Ref.^21^. In Refs.^22,23^ methods are developed through application of gaussian mixture model. In Ref.^24^ support vector machines is used for vehicle detection. SIFT algorithm is integrated with support vector machines in Ref.^25^ to achieve vehicle detection. For autonomous vehicles an object detection system is highlighted in Ref.^26^. Some R-CNN version of vehicle detection algorithms are discussed in Refs.^28,44,54^. Several significant YOLO based multi object vehicle detection algorithms are highlighted in Refs.^27^^,^^32–35,37–39,42^. Vehicle detection algorithms in different weather conditions are presented in Refs.^29,30,52^. Deep learning networks for vehicle detection are used in Refs.^31,36,41,43,45,46,55,56^. Several other notable works are available in Refs.^40,47–51,53,57^.

## Computational method

In this section, smart traffic vehicle management using Faster R-CNN based deep learning based ensemble method is highlighted. The research problem revolves around traffic management which involves vehicle segmentation at different background levels^5^. In order to achieve this, we study Faster R-CNN^5^ which analyses vehicles in smart traffic. The segmentation activity on various strategic areas of smart traffic analytics provide information related to decision-making activities. All methods were performed in accordance with the relevant guidelines and regulations by Samsung R&D Institute Delhi.

### Datasets used

**Figure 2:**
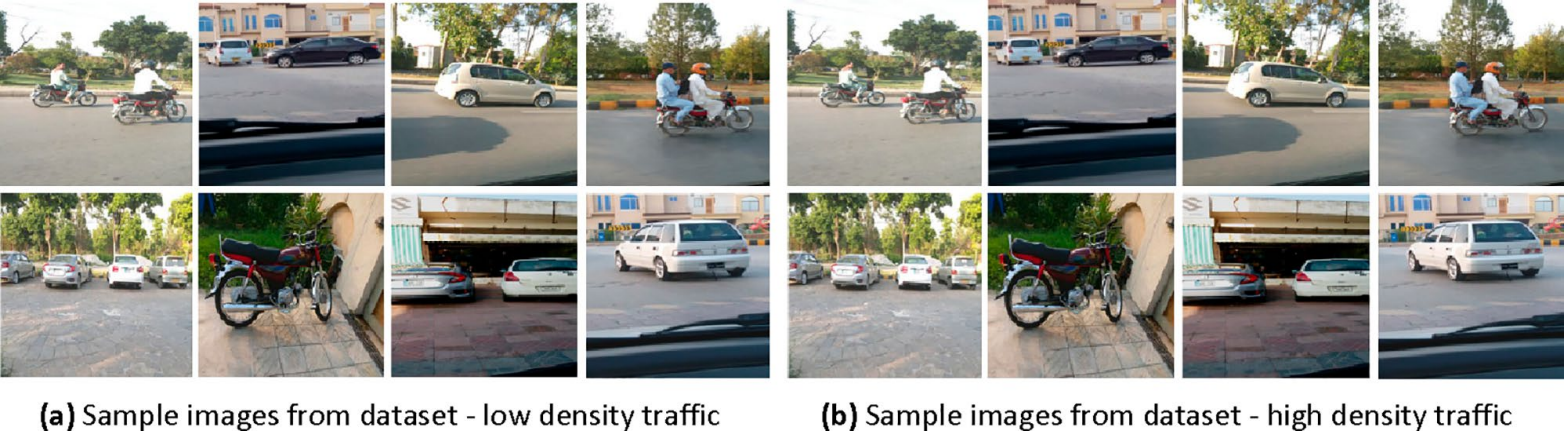
(**a**) Sample images from dataset—low density traffic (**b**) sample images from dataset—high density traffic.

The experimental datasets are adopted from Ref.^58^. The datasets are prepared keeping in view different traffic conditions. At the very first instance multi-class vehicle objects are considered. Several challenge factors such as traffic jams and overlapping vehicles are incorporated in dataset. Broadly speaking datasets are developed with respect to two different situations viz high density and low density traffic scenes. The former comes with several objects in single image while later has single class per image having no overlapping. In order to achieve better training, images from both above mentioned situations are placed in different datasets. The high density traffic scenes are considered from several places having traffic which are less crowded in nature. A total of 1000 images from six classes of vehicles viz two wheelers, three wheelers, four wheelers, six wheelers, eight wheelers and ten wheelers are developed. Figure 2a,b highlight some sample images. High density traffic scenes are considered from daily traffic which are congested in nature. 5000 images from above stated classes are created. Certain critical factors such as illumination, occlusions etc. are incorporated irrespective of appearance, shape, scale and size in this dataset. Table 1 highlights certain high and low density dataset statistics along with annotations of class. These datasets are preprocessed and augmented as discussed in “Data annotation and augmentation” section. Each of these datasets are benchmarked with respect to standard datasets^58^.

**Table 1:** Statistics of dataset images.

Dataset	Low density traffic	High density traffic
Source images	5000	1400
Annotations	50,595	2000
Classes		
Two wheeler	9000	120
Three wheeler	9351	200
Four wheeler	10,879	880
Six wheeler	10,500	600
Eight wheeler	5990	110
Ten wheeler	4875	90

### Data annotation and augmentation

**Figure 3:**
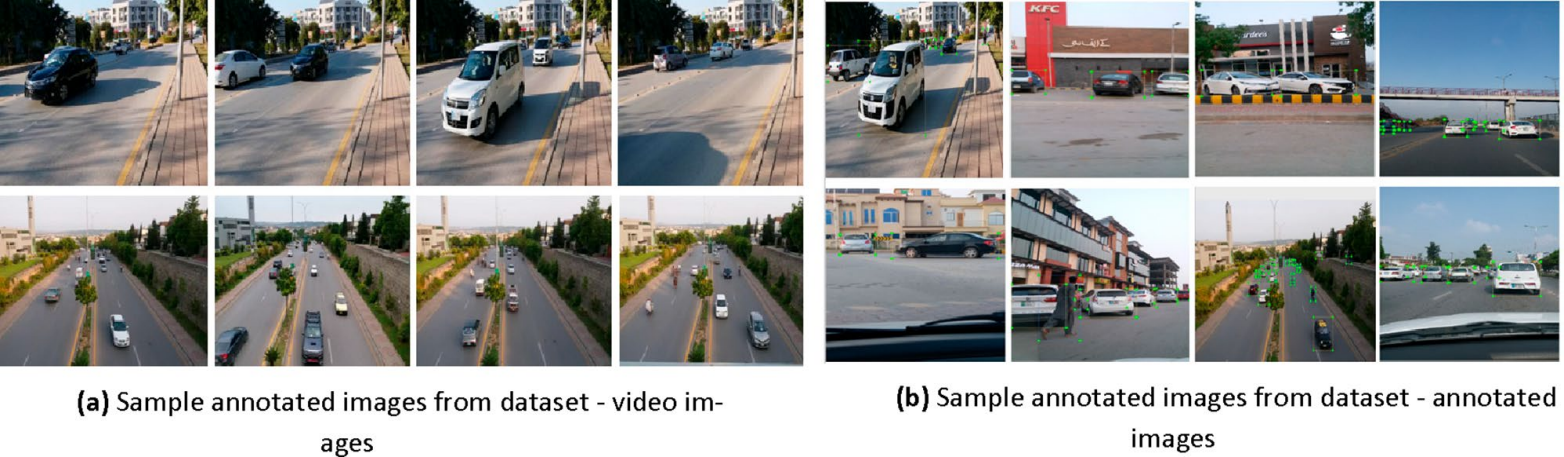
(**a**) Sample annotated images from dataset—video images (**b**) sample annotated images from dataset—annotated images.

In order to achieve reliable vehicle detection dataset classes are labelled. Based on motivation from Ref.^5^ an image tool^57^ has been used here towards labeling and annotation of this dataset. As shown in Fig. 3a,b for each object in image, a bounding box is assigned manually. In high density datasets (HDD) from high density traffic many bounding boxes are present. In low density datasets (LDD) from low density traffic few bounding boxes are present in single image. The label of respective class is specified by these bounding boxes. As mentioned in “Datasets used” section, the whole dataset is defined through six classes. In order to increase features of datasets such that better results are obtained data preprocessing and augmentation is used. These addresses various issues which can be present in images such as noise, inconsistency and unbalanced classes. The data augmentation process used here are in lines with discussed in Refs.^5,57^. Since augmented dataset has an unknown distribution it is benchmarked adhering to certain standards^58^.

### Faster R-CNN based method

Now we present a detailed description of proposed method. The method is highlighted considering four steps^5^ viz (a) minimization with adaptive background model (b) Faster R-CNN based subnet operation (c) Faster R-CNN initial refinement and (d) result optimization with extended topological active nets.

Adaptive background modeling is used to construct background with traffic video as input. The video frames are analyzed in order to develop background model. The objective here is to find best background estimate. With this on foreground model shadow and illumination change impacts are minimized. This process is initiated by first initializing few frames and then continuous updates are done each time. This leads to extraction of foreground from next set of frames. The initialization of background model is done considering first frame’s pixel values. A subsequent update of model is performed through calculation of value of pixel considering background model:

1 $$pixel_{j} \left( y \right) = \overline{pixel}_{j - 1} \left( y \right) + \frac{1}{{GP_{j} \sqrt {2\pi \sigma^{2} } }}\exp \left( {\frac{ - 1}{2}\left( {\frac{{y - y_{j} }}{\sigma }} \right)^{2} } \right)$$

Here $y_{j}$ represents value of pixel with respect to $j{\text{th}}$ frame. The value of $\overline{pixel}_{j}$ is computed as follows:

2 $$\overline{pixel}_{j - 1} \left( y \right) = \frac{{pixel_{j} \left( y \right)}}{{\mathop \sum \nolimits_{j} pixel_{j} \left( y \right)}}$$

Here number of frames considered is $N$. The gain parameter is denoted by $GP_{j}$. It takes care of background modeling’s learning rate. The new information is learned by increasing $GP_{j}$ as presented in Eq. (3) with prior information disappearing slowly.

3 $$GP_{j} = Gain\left( {\frac{2}{{1 + \exp \left( { - \frac{cont - \alpha }{\beta }} \right)}}} \right)$$

**Figure 4:**
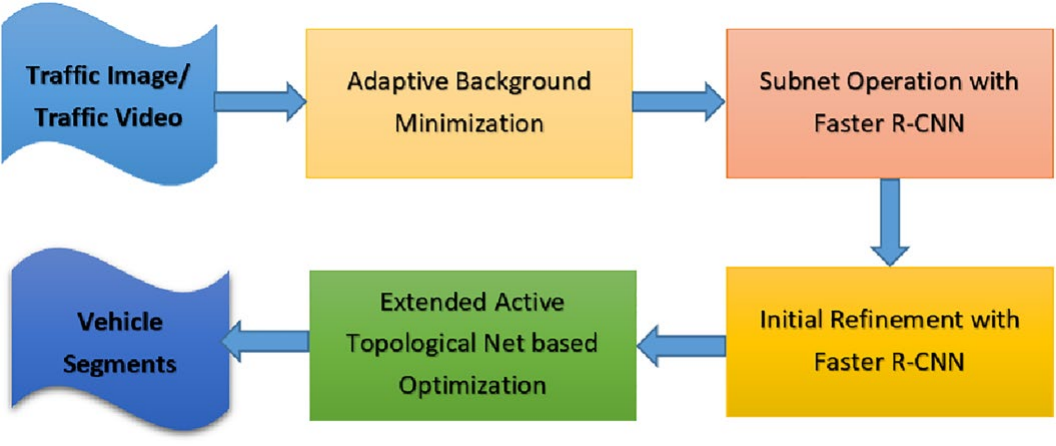
Architecture of vehicle segmentation method.

Here $Gain$ and $\alpha$ parameters take care of sinusoid function’s inflection point. The parameter $\beta$ controls gradient. The parameter $cont$ depends on number of frames. Background model is updated for every frame. Considering every fame with background as adaptive, after its subtraction we reach to foreground objects. After adaptive background model based minimization is performed, we present basic Faster R-CNN architecture used in this research. The architecture of Faster R-CNN is considered from Ref.^5^ having variation based baseline considered from Ref.^45^. The architecture is highlighted in Fig. 4. In order to validate proposed method, datasets mentioned in “Datasets used” section are used.

The convolutional feature map is developed when entire image is processed convolutional and max pooling layers. For each object’s RoI pooling layer, feature vector of fixed length is extracted. The input of sequence of fully connected layers are feature vectors. From a set of fully connected layers, output passes to sibling layers where softmax probability estimates are produced. The object classes estimate softmax probability. Here background class with set of other layers are considered. The encoding is performed on set of values with respect to refined positions in bounding box for each object class. For RoI pooling layer we use max pooling. This allows conversion of features into small feature map. Here convolutional feature map based RoI is defined through 4-tuple. RoI max pooling divides rectangular window into sub window grids. The channel independent pooling is applied for each feature map. The network weights are trained through backpropagation in Faster R-CNN. A hierarchical sampling of RoIs is performed for each image. Stochastic gradient descent (SGD) training for Faster R-CNN is done in mini-batches. For training with larger datasets, execution of SGD is done for more iterations. Faster R-CNN makes use of sibling output layers. For each RoI we have discrete probability distribution as initial output. The probabilities for fully connected layer have outputs as softmax. The labeling for RoI is performed with respect to ground truth class. For each training bounding box regression having ground truth is considered. For each labeled RoI considering multitask loss, joint classification is present with respect to training and bounding-box regression. The optimization of multitask loss is performed as highlighted in Ref.^5^. For whole image classification, convolutional layers’ calculation time is greater than fully connected layers. RoIs processing time is appreciably large for detection.

Now subnet operation with Faster R-CNN is presented. The foreground image considers a mesh. This is moved towards subnet operation for Faster R-CNN. The training image dataset is adopted from Ref.^19^. This is marked with respect to active net grids. The subnet is represented is binary matrix with 1 as indicator for link presence of mesh and 0 as indicator for link absence. Here Faster R-CNN is trained where features are learned in order to produce ground truth specific results. The U-Net based CNN Ref.^59^ helps us to perform initial subnet operation. The extended topological active net refines initial subnet. Faster R-CNN produces hole based mesh considering all background. The objects have mesh nodes. Those mesh nodes which where boundary is not crossed are removed. It is reached though extended topological active net energy minimization. The energy function used here is highlighted in Eq. (4). By using Eq. (1) adaptive background model is developed.

4 $$Energy\left( {w\left( {a,b} \right)} \right) = \mathop \smallint \limits_{0}^{1} \mathop \smallint \limits_{0}^{1} \left[ {Energy_{internal} \left( {w\left( {a,b} \right)} \right) + Energy_{external} \left( {w\left( {a,b} \right)} \right)} \right]dadb$$

The mesh deformation is done using greedy approach. In situations with complex deformation of shapes, extended topological active net produces different energy term. This it achieves through various thresholds. When clutters and occlusion are present in vehicle image segmentation extended topological active net always reaches local minima. This results in low segmentation accuracy. The improved version of extended topological active net provides resolution to these problems. The combined effect of all models used here leads us to better results.

### Training process

The training process is now briefly discussed. The annotated and augmented data is trained using Faster R-CNN algorithm. In order to perform training, several parameters like size of batch, epochs needed and resolution of image are used. Since network is trained from scratch, random weight initialization is performed. Here initially trained COCO weights^5^ are used towards model training with appreciable time and computation benefits. The best weight values are obtained using initially trained Faster R-CNN having transfer learning. Chaudhuri ^5^ datasets are used as benchmark in order to train stated custom datasets. The batch sizes of 5, 10, 20, 30 and 40 are considered. The epochs are also changed to 100, 200, 300, 400 and 500. The confidence values are considered between 0.4 and 0.6. The best weights are used to detect objects in datasets. The predicted labels and assessment images incorporating bounding boxes with confidence values are also obtained.

### Evaluation criteria

Some of the significant metrics in evaluation of smart cities include^5^ key process indicators alignment with respect to several community priorities spanning neighborhoods, alignment of investment with respect to community priorities, investment efficiency, information flow density, infrastructure services and community benefits inherent quality. In this research various evaluation metrics^5^ are used for measuring performance of our method which are discussed in “Experiments and results” section. These metrics help to identify efficiency and robustness of proposed method. Here, mean average precision (mAP) is calculated for recall values lying between 0 and 1. Along with this some comparative analysis of results is also performed.

### Ethical approval and informed consent

Author has ethical and informed consent for data used in this research.

## Experiments and results

Here a detailed discussion on simulation results is presented. We conducted detailed experiments in Google Colab having T4 GPU with Intel Xeon CPU and 64 GB of RAM. Python version 3.11.5 has been used as simulation tool in this research. In order to assess vehicle detection method performance, several state-of-the-art detectors are evaluated. Also various comparisons are performed with stated method considering accuracy and execution times. All methods are trained on data adapted from COCO^60^ and DAWN^61^ datasets.

**Figure 5:**
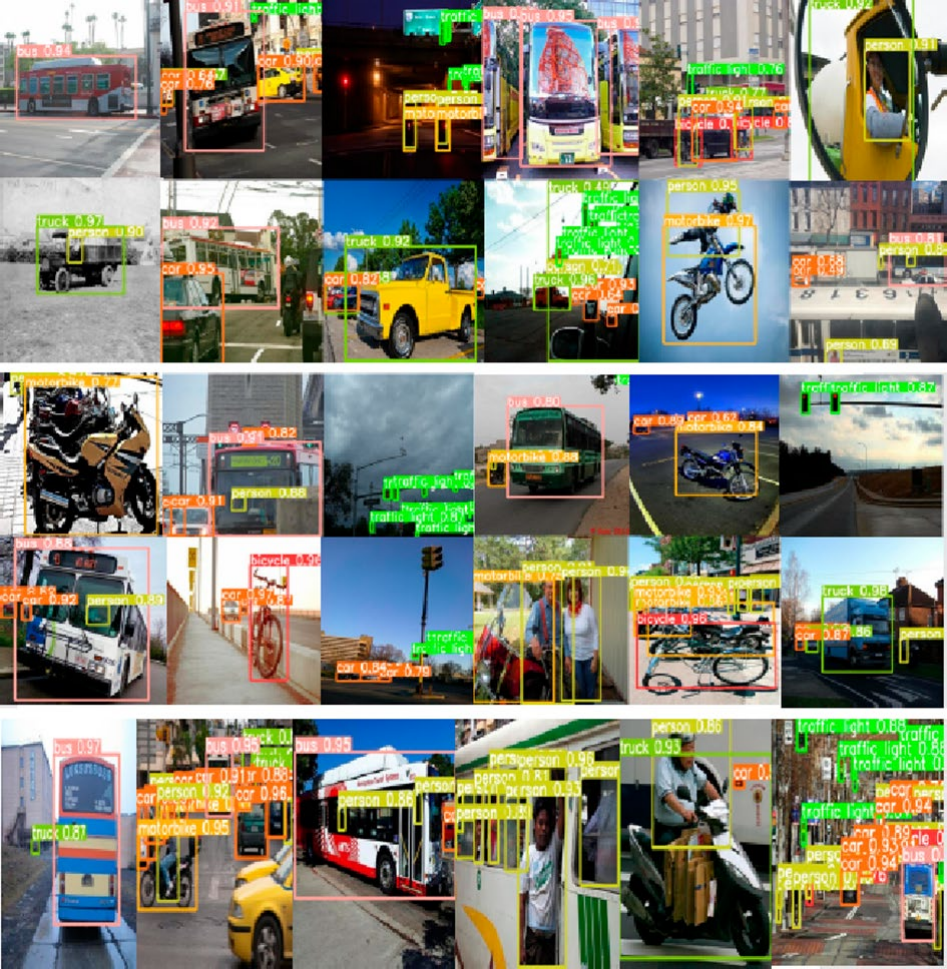
COCO dataset vehicle detection results.

The detection and segmentation of objects is performed by COCO dataset considering natural contexts^5^. In Table 2 COCO dataset highlights several objects collected from complex scenes. The dataset has images of 100 different object types with 3 million instance labels. The results are highlighted in Fig. 5. Certain YOLOv5 semantics are adopted from Ref.^57^. All vehicles are accurately detected by stated method considering variation in illumination. In order to further validate superiority of stated method we perform a comparative analysis with 14 methods as presented in Table 3. Some significant observations are briefly highlighted here. For images with different resolutions, stated computational structure provides best performance in terms of mAP values. The methods presented in Refs.^49,50,55,56^ also provide promising results with respect to COCO datasets. He et al.^54^ makes use of ResNet and produces results on lower side for object detection in COCO datasets. Similar results are obtained from methods presented in Refs.^45,47^. Here miscellaneous objects are detected on COCO datasets with promising results. These include vehicles of varying shapes and sizes. All results are highlighted in Fig. 5.

**Table 2:** Certain dataset specific statistics.

Dataset	Number of classes	Training	Validation	Testing
HDD	6	16,799	20,575	20,685
LDD	6	80,577	10,879	10,780
COCO	90	300,000	18,000	12,000

*HDD* high density dataset from high density traffic, *LDD* low density dataset from low density traffic.

**Table 3:** COCO dataset accuracy comparison.

Computation backbone	Input size	Multi-scale	mAP (%)
CSPDarkNet53^42^	512 × 512	False	48.64
CNN^43^	512 × 512	False	49.00
R-CNN^44^	512 × 512	False	47.40
BottlenectCSP^45^	512 × 512	False	28.99
VGGNet-16^47^	512 × 512	False	30.40
ResNet-101-FPN^48^	512 × 512	False	40.40
VGGNet-16^49^	800 × 800	False	42.00
ResNet-101^50^	800 × 800	False	49.40
ResNet-101^51^	512 × 512	False	40.50
CNN + SVM^52^	512 × 512	False	50.10
bn + relu^53^	512 × 512	False	34.90
ResNet-C4-FPN^54^	512 × 512	False	32.88
ResNet-50^55^	512 × 512	False	50.90
SiNet^56^	512 × 512	False	51.50
CSF^57^	512 × 512	False	52.45
Our method	512 × 512	False	58.90

**Figure 6:**
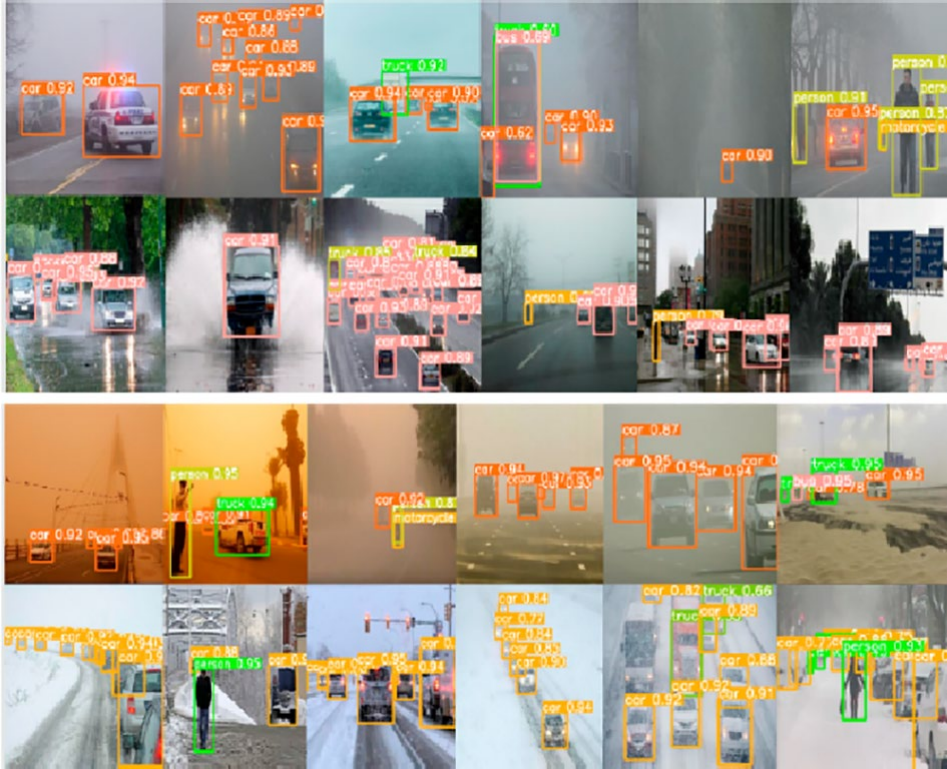
Vehicle detection results on DAWN datasets.

The DAWN dataset is used in order to study and investigate performance of stated method. DAWN dataset has 2000 image with different variations^5^. It shows varying traffic situations in different weather conditions. Figure 6 shows fairly detailed results considering significant observations. The stated method addresses all prevailing weather situations such as rainy days, normal dry days and snowy days. A comparison is performed with results presented in Table 4. Some observations are highlighted here. In fog situation^54^ has highest rank. Our method exceeds results of Ref.^54^. Here Refs.^43,52^ have lowest ranks. In rain situation our method produces best results followed by Refs.^45,48,50,51,57^. Here Ref.^52^ has lowest rank. In snow situation our method has highest rank followed by Refs.^51,57^. Here Refs.^45,47,50,51^ produce similar results. In dry day situation stated method achieves highest rank. Here Refs.^42,48,49^ yield similar results. Min et al.^52^ has lowest rank.

**Table 4:** Comparison of methods on DAWN datasets.

Computation backbone	Fog	Rail	Snow	Dry day
CSPDarkNet53^42^	26.40	31.55	39.95	24.10
CNN^43^	24.00	21.10	38.32	23.80
r-cnn^44^	27.20	21.30	28.30	18.00
BottlenectCSP^45^	29.31	41.21	43.00	24.02
VGGNet-16^47^	23.40	24.60	37.90	15.83
ResNet-101-FPN^48^	28.95	41.10	43.00	24.09
VGGNet-16^49^	23.10	27.65	34.00	24.10
ResNet-101^50^	29.70	40.10	43.00	23.99
resnet-101^51^	28.10	40.40	43.02	24.10
CNN + SVM^52^	16.50	14.08	15.38	10.69
bn + relu^53^	25.08	19.14	23.18	17.38
FtesNet-C4-FPN^54^	29.68	30.32	33.93	24.00
ResNet-50^55^	28.83	27.68	30.19	24.03
SiNet^56^	26.45	20.09	27.92	11.31
CSP^57^	29.66	41.21	43.01	24.14
Our method	30.40	45.55	45.79	25.60

**Figure 7:**
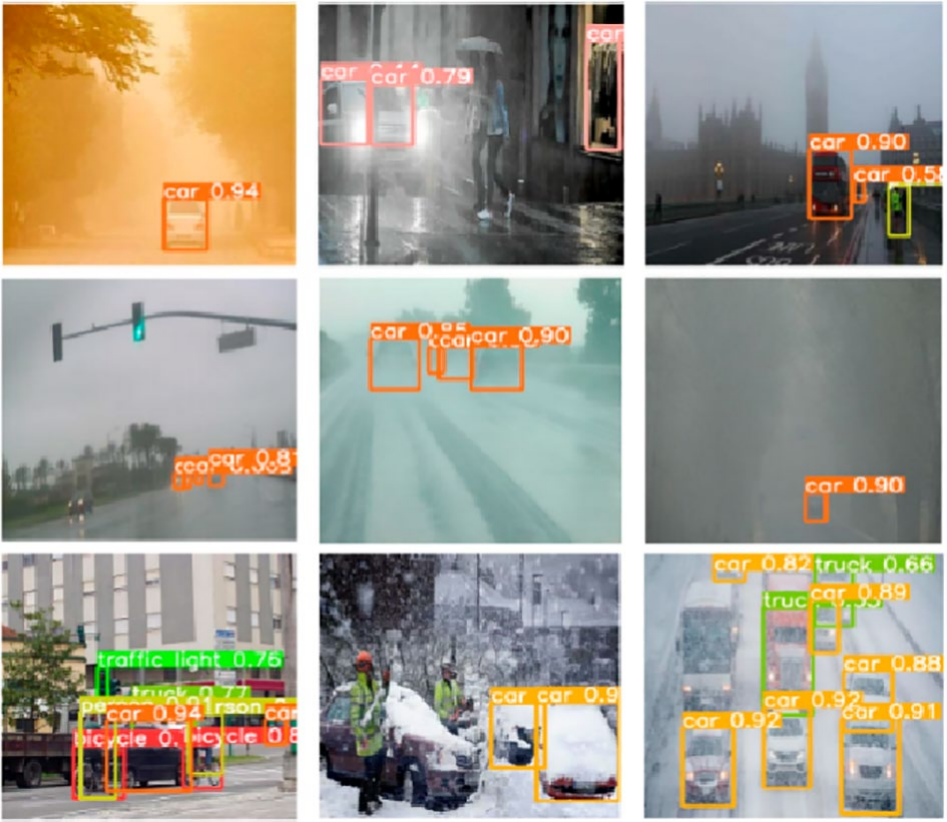
Vehicle detection method on sample image.

Now we present some additional insights in this research with respect to diverse range of environments. All state-of-the-art object detection methods have been studied in this research. These results are briefly discussed here. In Ref.^42^ BIT-Vehicle and UADETRAC datasets are investigated. In Ref.^57^ 3 different datasets are studied which several variations with respect to road conditions, weather as well as complex background. In Refs.^43,48^ primary concentrations are on KITTI and DAWN datasets with certain deviations which produces appreciable results. In Ref.^44^ investigation of object detection capability is performed on COCO and DAWN datasets. In Ref.^45^ CARLA dataset is explored. The results presented here extends method's detection capability to 3 other datasets. PASCAL VOC 2007 dataset is studied in Refs.^47,51^. In Ref.^37^ PASCAL dataset is studied which contains annotated images of various objects. Here detection capability of method is expanded to 3 different vehicle datasets. In Refs.^49,50,53,54^ COCO and DAWN datasets are used in order to validate methods for object detection. In Ref.^54^ various object detection methods are presented for detection of different objects. PETS2009 and changedetection.net 2012 datasets are studied in Ref.^52^ with DLR Munich vehicle and VEDAI datasets in Ref.^55^ and KITTI and LSVH datasets in Ref.^56^. The vehicle detection methods on these datasets produce appreciable results. In DAWN dataset has more challenging images with fog, rain, dry day and snow. However, as shown in Fig. 7 very low vehicle detection results are achieved. But our method performs well in this situation. Considering several state-of-the-art approaches, a detailed comparison of is performed alongwith our method. It is seen that in different situations our method performs well. In COCO dataset our method produces appreciable results in comparison to other methods.

**Figure 8:**
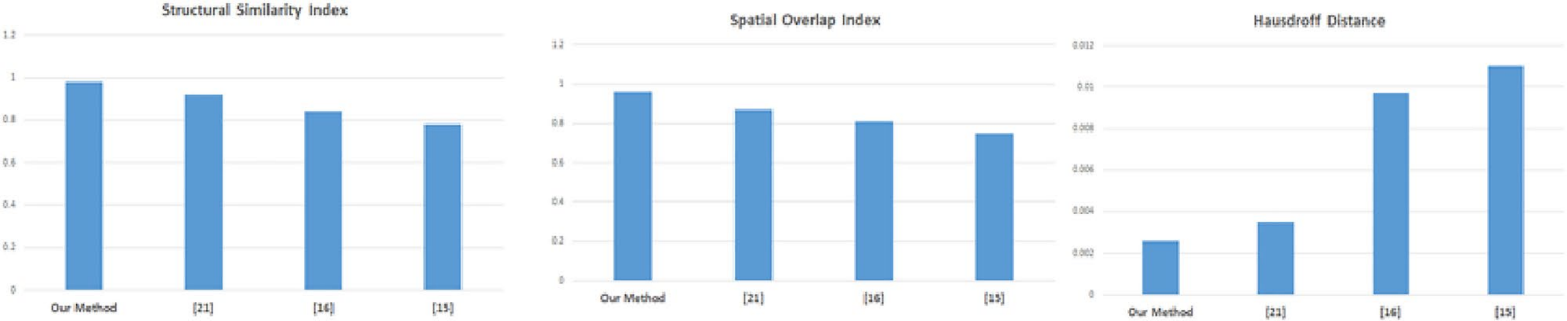
Comparative performance of our method considering structural similarity index, spatial overlap distance and hausdroff distance.

The metrics for Type I and Type II errors account for false positives and false negatives respectively. The Table 5 highlight results with respect to Type I and Type II errors for HDD, LDD, COCO and DAWN datasets. The illustrative loss functions are applied to Type I and Type II errors. A loss function is imposed when accuracy report is chosen. When loss function is minimized accuracy increases. As a result of this care needs to be enforced when using loss functions. Here loss is reduced through minimization of objective function^5^ which is weighted sum of localization and confidence losses^5^. The localization loss is smooth L1 loss between true values and predicted bounding box correction. The coordinate correction transformation is identical to R-CNN in bounding box regression. The confidence loss represents how likely an object is contained in bounding box. It is calculated using logistic regression function based on intersection over union (IoU) between predicted bounding box and ground truth bounding box. These results are further strengthened with accuracy, specificity, sensitivity (recall), precision and F1-score values in Table 6. In order to highlight significance of results more, Fig. 8 represents comparative performance of our method with respect to structural similarity index, spatial overlap distance and hausdroff distance^5,15,16,21^ metrics.

**Table 5:** Type I and type II errors for HDD, LDD, COCO and DAWN datasets.

Dataset	Classes	Validation	Errors in validation		Testing	Errors in testing	
			Type I	Type II		Type I	Type II
HDD	6	20575	277	200	20,685	335	236
LDD	6	10,879	176	160	10,780	184	175
COCO	90	18,000	265	180	12,000	207	205
DAWN	4	12,879	187	179	12,790	189	177

**Table 6:** Achieved results of our method with accuracy, specificity, sensitivity, precision and F1-score performance metrics for HDD, LDD, COCO and DAWN datasets.

Dataset	Performance metrics				
	Accuracy	Specificity	Sensitivity	Precision	Fl-Score
HDD	0.9724	0.9770	0.9679	0.9678	0.9725
LDD	0.9666	0.9662	0.9672	0.9670	0.9679
COCO	0.9656	0.9646	0.9666	0.9667	0.9668
DAWN	0.9714	0.9713	0.9714	0.9715	0.9723

## Conclusion

In this research work smart traffic management of vehicles is studied using Faster R-CNN based deep learning method. It is an intractable problem in computer vision and artificial intelligence domain. When occlusions, background with clutters and traffic with density variations are present, this problem becomes more challenging. The computational paradigm involves four steps viz minimization with adaptive background model, Faster R-CNN based subnet operation, Faster R-CNN initial refinement and result optimization with extended topological active nets. The concept of adaptive background modeling is incorporated in this framework. The shadow and illumination related issues are also addressed. The topological active net deformable models help to achieve higher segmentation accuracy. The deformations are reached with topological and extended topological active nets. Mesh deformation helps in minimization of energy. The segmentation accuracy is improved with modified version of extended topological active net. The superiority of this method in comparison to other methods is highlighted with experimental results. This achieved using different performance metrics such as Type I (for false positives) and Type II (for false negatives) errors, accuracy, specificity, sensitivity (recall), precision and F1-score values. The results are made more significant through comparative performance of our method with other methods with respect to structural similarity index, spatial overlap distance and hausdroff distance metrics.

## Data Availability

The datasets used and/or analysed during the current study available from the corresponding author on reasonable request. All data used in this research has been developed at Samsung R & D Institute New Delhi India as mentioned in Reference 58. The Institution does not allow to share and provide access to research data to public domains. In view of this, data used in this research cannot be shared. However, two more datasets COCO and DAWN used in this research are highlighted in References 60 and 61 can be shared and accessed.
